# Sleep disturbance in children with attention-deficit hyperactivity disorder: A systematic review

**DOI:** 10.5935/1984-0063.20190088

**Published:** 2019

**Authors:** Renata Martins, Janaina Cristina Scalco, Geraldo Jose Ferrari Junior, Juliana Gonçalves da Silva Gerente, Matheus da Lapa Costa, Thaís Silva Beltrame

**Affiliations:** 1 MSc. In Physiotherapy at Santa Catarina State University, Florianópolis/SC/Brazil.; 2 MSc.in Human Movement Science at Santa Catarina State University, Florianópolis/SC/Brazil.; 3 MSc.in Rehabilitation in Visual Disabilities at Faculty of Human Kinetics, Lisbon/Portugal.; 4 BSc. in Physical Education at Santa Catarina State University, Florianópolis/SC/Brazil.; 5 Professor Dr. at Physical Education Department and Post-Graduation Program in Human Movement Science at Santa Catarina State University, Florianópolis /SC/ Brazil.

**Keywords:** Attention Deficit Disorder with Hyperactivity, Sleep Wake Disorders, Child

## Abstract

The aim of the present systematic review was to compare sleep disorders in children, from 7 to 12 years old, with and without an attention-deficithyperactivity disorder (ADHD) diagnosis. Electronic literature search of PubMed, LILACS, Scopus, Web of Science and Cochrane Library databases was conducted in September 2017. We included cross-sectional observational studies comparing the sleep of children between 7 and 12 years old, with and without an ADHD diagnosis, reported according to the Diagnostic and Statistical Manual of Mental Disorders criteria. The studies with other research designs, those that included adolescents and/or adults in the sample and those who evaluated the sleep of children with ADHD and other associated comorbidities were excluded. A total of 1911 articles were identified. After analyzing, 8 articles were compatible with the theme and included in the review. For sleep evaluation, most of the studies used an objective measure together with another subjective measure. Three out of six studies that used objective measures did not observe any differences between children with and without ADHD diagnosis. Children with ADHD presented more sleep disturbances when compared to children without the diagnosis. These disorders were diverse, yet inconsistent among the surveys. More studies are needed to clarify and for robust results.

## INTRODUCTION

The attention-deficit hyperactivity disorder (ADHD) is one of the most common disorders of childhood and adolescence, characterized as a neurodevelopmental disorder that impairs the personal, social and academic performance^1^. The etiology of ADHD is not well established yet. Literature shows, however, a wide influence of heritability and mere suspicion of environmental factors on its development^2^^,^^3^. Although neurological influence and genetic researches point out that people with ADHD have dysfunction of dopaminergic neurotransmitters in the frontal, subcortical and limbic brain regions^4^, the diagnosis of this disorder, generally identified in school age, is possibly obtained by detailed clinical history and interviews with parents and teachers about the child’s behavior^5^.

The most commonly used tool in clinical practice for the diagnosis of ADHD is the Diagnostic and Statistical Manual of Mental Disorders (DSM-5^®^)^1^. According to DSM-5 criteria, the symptoms of ADHD can be divided into three subtypes based on its predominance: hyperactive-impulsive, inattentive and combined. These characteristics must be expressed in two different places, usually at home and at school, for at least six months^1^.

Besides that, literature reports that sleep problems are common among children with ADHD, even in the absence of stimulating medication^6^^,^^7^. In this context, Biederman & Spencer^8^ reported changes in noradrenergic and dopaminergic neurotransmitter pathways in individuals with ADHD, which are also found in patients with sleep disorders. Thus, sleep disorders and ADHD may have similar symptoms, and it is possible that patients with sleep disorders may be misdiagnosed with ADHD and *vice versa*^9^.

To evaluate the sleep of children with ADHD, objective measures such as videotaping, polysomnography, and actimetry are usually applied, as well as subjective measures such as questionnaires for parents about the child’s sleeping habits^10^. The identification and management of sleep problems in children with ADHD are extremely important since these problems might cause suffering to children and their parents and even exacerbate the symptoms of ADHD, which implies a lower quality of life^11^^,^^12^.

In considering the latter information, this research aimed to conduct a systematic literature review to compare sleep changes in 7 to 12-year-old children with and without an ADHD diagnosis.

## METHODS

## Protocol and registration

This systematic review has adhered to the reported items on recommendation *Preferred Reporting Items for* Systematic Reviews and Meta-analyses (PRISMA)^13^. The study protocol was registered on the International Prospective Register of Systematic Reviews (PROSPERO) platform with the number CRD42018107709.

## Eligibility criteria

### Inclusion criteria

Cross-sectional observational studies comparing the sleep of 7 to 12-year-old children, with and without an ADHD diagnosis were included. They were reported according to DSM-IV criteria and/or medical diagnosis, full articles available in English, Spanish and/or Portuguese, with no set limitation on publication dates.

It justifies the age range delimited by the objective that this revision serves the studies in school scope, so, from the age of seven, the elementary school begins, at which point the characteristics of ADHD can appear and make the process difficult of teaching and learning. Moreover, although the transition from the infantile phase to adolescence is subjective due to maturation, there are several cut-off points that delimit these phases, in order to obtain greater comparability, so we chose 12 years, since we believe that older students would have higher probability of being in more advanced stages of maturation, which would interfere in the sleep analyzes^14^.

### Exclusion criteria

The following exclusion criteria were applied: 1 - reviews, clinical trials, editorial letters, studies involving animals; abstracts published in Annals of events; 2 - studies that included adolescents and/or adults in the sample; 3 - studies that evaluated the sleep of children with ADHD and other associated comorbidities; 4 - studies that included medicated children in the data collected.

### Sources of information and search strategy

The research strategies were individually developed for each of the following electronic databases: PubMed, LILACS, Scopus, Web of Science and Cochrane Library based on PECOS approach. Our strategy was directed at the Population (e.g. children, preschoolers), Exposure (ex. Attention Deficit/Hyperactivity Disorder), and Outcomes (ex. sleep disorders, polysomnography). Further research on the gray literature was done by visiting Google Scholar. The Descriptors in Health Science (DeCS) and Medical Subject Headings (MeSH) were the base for our selection of descriptors (the complete search strategy is in Table 1).

**Table 1:** Search strategy used for each database.

**PubMed**	((("child"[MeSH Terms] OR "child" OR "children" OR "preschool children" OR "school age" OR "kids" OR "pediatrics"[MeSH Terms] OR "pediatrics"[Title/Abstract] OR "pediatric"[Title/Abstract])) AND ("sleep wake disorders"[MeSH Terms] OR "sleep wake disorder" OR "disorder, sleep wake" OR "disorders, sleep wake" OR "wake disorder, sleep" OR "wake disorders, sleep" OR "sleep disorder" OR "sleep disorders" OR "disorder, sleep" OR "disorders, sleep" OR "Sleep Apnea, Obstructive"[MeSH Terms] OR "apneas, Obstructive Sleep" OR "Obstructive Sleep Apneas" OR "Sleep Apneas, Obstructive" OR "Obstructive Sleep Apnea Syndrome" OR "Obstructive Sleep Apnea" OR "OSAHS" OR "Syndrome, Sleep Apnea, Obstructive" OR "Sleep Apnea Syndrome, Obstructive" OR "Apnea, Obstructive Sleep" OR "OSA")) AND ("attention deficit disorder with hyperactivity"[MeSH Terms] OR "attention deficit disorders with hyperactivity" OR "attention deficit hyperactivity disorders" OR "attention deficit-hyperactivity disorder" OR "attention deficit-hyperactivity disorders" OR "deficit-hyperactivity disorder, attention" OR "deficit-hyperactivity disorders, attention" OR "disorder, attention deficit-hyperactivity" OR "disorders, attention deficit-hyperactivity" OR "ADHD" OR "ADDH" OR "attention deficit hyperactivity disorder")
**LILACS**	"Child" OR "Criança" OR "Niño" OR "Children" OR "Crianças" OR "Niños" OR "Child, Preschool" OR "Preescolar" OR "Pré-Escolar" OR "Pediatrics" OR "Pediatría" OR "Pediatria" [Palavras] AND "sleepwakedisorders" OR "transtornos de sono-vigília" OR "trastornosdelsueño-vigilia" OR "sleepdisorders" OR "distúrbios do sono" OR "trastornosdelsueño" OR "sleepdisorder" OR "distúrbio do sono" OR "trastornodelsueño" OR "Obstructivesleepapnea" OR "apneia obstrutiva do sono" OR "Apnea obstructiva del sueño" [Palavras] AND "attention déficit disorderwithhyperactivity" OR "transtorno do déficit de atenção com hiperatividade" OR "trastorno por deficit de atención com hiperactividad" OR "transtorno de hiperatividade e falta de atenção" OR "Transtorno de hiperatividade e déficit de atenção" OR "transtorno de falta de atenção com hiperatividade" OR "ADDH" OR "ADHD" OR "TDAH"
**Scopus**	(TITLE-ABS-KEY("child" OR "child" OR "children" OR "preschool children" OR "school age" OR "kids" OR "pediatrics" OR "pediatrics" OR "pediatric") AND TITLE-ABS-KEY("sleep wake disorders" OR "sleep wake disorder" OR "disorder, sleep wake" OR "disorders, sleep wake" OR "wake disorder, sleep" OR "wake disorders, sleep" OR "sleep disorder" OR "sleep disorders" OR "disorder, sleep" OR "disorders, sleep" OR "Sleep Apnea, Obstructive" OR "apneas, Obstructive Sleep" OR "Obstructive Sleep Apneas" OR "Sleep Apneas, Obstructive" OR "Obstructive Sleep Apnea Syndrome" OR "Obstructive Sleep Apnea" OR "OSAHS" OR "Syndrome, Sleep Apnea, Obstructive" OR "Sleep Apnea Syndrome, Obstructive" OR "Apnea, Obstructive Sleep" OR "OSA") AND TITLE-ABS-KEY("attention deficit disorder with hyperactivity" OR "attention deficit disorders with hyperactivity" OR "attention deficit hyperactivity disorders" OR "attention deficit-hyperactivity disorder" OR "attention deficit-hyperactivity disorders" OR "deficit-hyperactivity disorder, attention" OR "deficit-hyperactivity disorders, attention" OR "disorder, attention deficit-hyperactivity" OR "disorders, attention deficit-hyperactivity" OR "ADHD" OR "ADDH" OR "attention deficit hyperactivity disorder"))
**Web of** **Science**	Topic=("child" OR "child" OR "children" OR "preschool children" OR "school age" OR "kids" OR "pediatrics" OR "pediatrics" OR "pediatric") AND Topic=("sleep wake disorders" OR "sleep wake disorder" OR "disorder, sleep wake" OR "disorders, sleep wake" OR "wake disorder, sleep" OR "wake disorders, sleep" OR "sleep disorder" OR "sleep disorders" OR "disorder, sleep" OR "disorders, sleep" OR "Sleep Apnea, Obstructive" OR "apneas, Obstructive Sleep" OR "Obstructive Sleep Apneas" OR "Sleep Apneas, Obstructive" OR "Obstructive Sleep Apnea Syndrome" OR "Obstructive Sleep Apnea" OR "OSAHS" OR "Syndrome, Sleep Apnea, Obstructive" OR "Sleep Apnea Syndrome, Obstructive" OR "Apnea, Obstructive Sleep" OR "OSA") AND Topic=("attention deficit disorder with hyperactivity" OR "attention deficit disorders with hyperactivity" OR "attention deficit hyperactivity disorders" OR "attention deficit-hyperactivity disorder" OR "attention deficit-hyperactivity disorders" OR "deficit-hyperactivity disorder, attention" OR "deficit-hyperactivity disorders, attention" OR "disorder, attention deficit-hyperactivity" OR "disorders, attention deficit-hyperactivity" OR "ADHD" OR "ADDH" OR "attention deficit hyperactivity disorder")
**Cochrane Library**	"child" or "child" or "children" or "preschool children" or "school age" or "kids" or "pediatrics" or "pediatrics" or "pediatric" AND "sleep wake disorders" or "sleep wake disorder" or "disorder, sleep wake" or "disorders, sleep wake" or "wake disorder, sleep" or "wake disorders, sleep" or "sleep disorder" or "sleep disorders" or "disorder, sleep" or "disorders, sleep" or "Sleep Apnea, Obstructive" or "apneas, Obstructive Sleep" or "Obstructive Sleep Apneas" or "Sleep Apneas, Obstructive" or "Obstructive Sleep Apnea Syndrome" or "Obstructive Sleep Apnea" or "OSAHS" or "Syndrome, Sleep Apnea, Obstructive" or "Sleep Apnea Syndrome, Obstructive" or "Apnea, Obstructive Sleep" or "OSA" AND "attention deficit disorder with hyperactivity" or "attention deficit disorders with hyperactivity" or "attention deficit hyperactivity disorders" or "attention deficit-hyperactivity disorder" or "attention deficit-hyperactivity disorders" or "deficit-hyperactivity disorder, attention" or "deficit-hyperactivity disorders, attention" or "disorder, attention deficit-hyperactivity" or "disorders, attention deficit-hyperactivity" or "ADHD" or "ADDH" or "attention deficit hyperactivity disorder"

All searches were conducted in September 2017. In addition, manual searches were conducted in the reference lists of included studies. A reference management software (EndNote^®^, Thomson Reuters, Philadelphia) was used to collect references and exclude duplicates.

### Selection of studies

Two stages were conducted to select the studies. In stage one, the titles and abstracts identified in all databases were examined independently by two reviewers. We excluded those who did not meet the pre-established selection criteria. In stage two, the same reviewers evaluated the full texts of the selected studies. In cases of divergence related to the exclusion of a title, a third reviewer analyzed the abstract or full text.

### Data extraction process

An original datasheet was used to collect the necessary information from the selected studies. Two authors independently collected the information. All the controversies in that process were discussed and decided by a third author to ensure accuracy in the recorded information. The collected data consisted of the characteristics of the study (authors, publication year), the population (sample size, the age of participants, gender), characteristics of sleep evaluation methods, and characteristics of the results (comparative measures between groups with and without ADHD).

### Evaluation of selected studies

An adaptation of the STROBE (*Strengthening the reporting of observational studies in epidemiology*) check-list was used to evaluate the selected studies, which gathers recommendations to improve the methodological quality of observational studies^15^, assigning scores to characterize the studies. The checklist consists of 14 items, which are stratified or not in sub-items, covering a total of 22 items. Each of the items and sub-items received a proportional score, totaling a maximum sum of 20 points^16^. The two authors involved in the studies selection independently conducted this evaluation. All disagreements were worked out with the assistance of a third author.

### Summarization measures

Average percentage and “*p*” values expressed the results related to the presence of sleep changes in children with ADHD. The results of the studies that did not present these values were demonstrated according to the parameters of the own study.

## RESULTS

**Figure 1:**
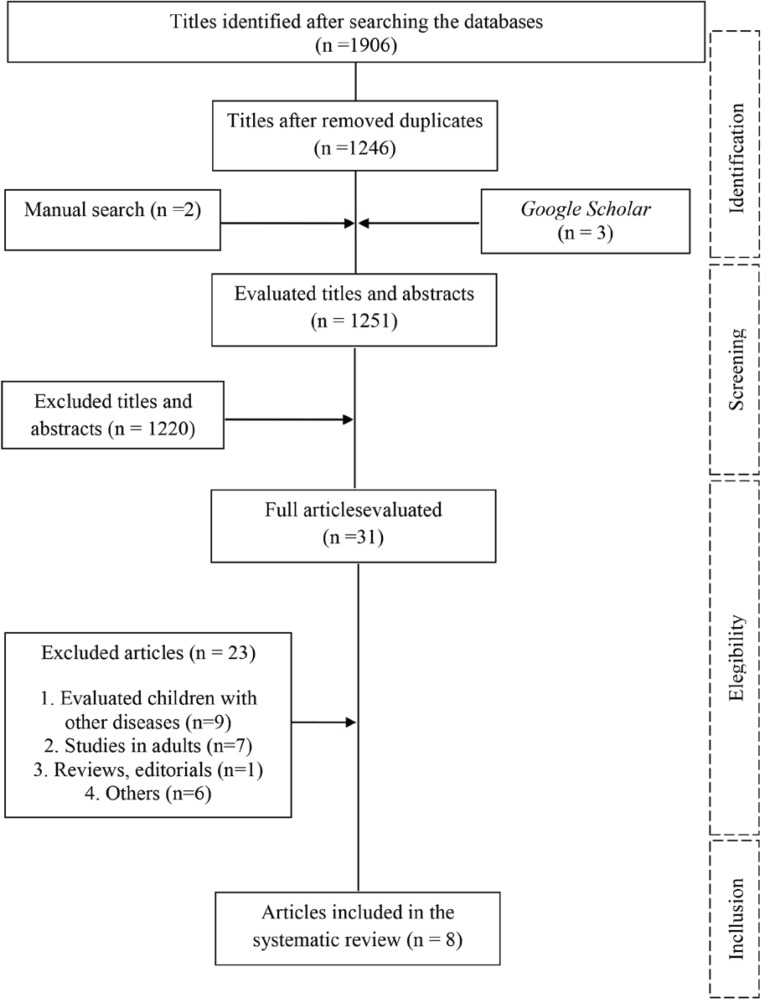
Flow diagram of the studies selection.

A total of 1911 articles were identified through databases. After removing duplicates, 1249 articles were sent for title and abstract selection in pairs. Lastly, 30 were sent for final analysis, where the full article was read. We analyzed two studies (one of them was excluded for its abstract), including a manual search in the reference lists of the selected articles.Ultimately, eight articles were included in this review. Figure 1 shows the diagram that illustrates article selection and exclusion steps.

### Characteristics of the studies

Three of the cross-sectional observational studies included were conducted in Canada^17^^,^^18^, two in Korea^19^^,^^20^, two in Turkey^21^^,^^22^and one in Mexico^23^. The sample size of the studies ranged from 15 to 156 children with ADHD and from 15 to 111 children without the disorder. The total of eight studies included 366 children with ADHD and 333 children without it.

Regarding the sleep evaluation method, most of the studies used an objective measure in combination with another subjective measure^17^^-^19^,^^21^^,^^24^. Seven studies used questionnaires^17^^-^22^,^^24^, four applied polysomnography^18^^,^^19^^,^^21^^,^^24^ and two of them, actimetry^17^^,^^20^.

### Synthesis of results

When checking the results, the ADHD group had worse sleep conditions, with more difficulties, greater perception of disorders when compared to the control group, except for sleep duration. The subjective duration of sleep presented an inconsistent result, which Gruber’s study^18^ found lower sleep duration in the ADHD group, unlike other studies^17^^,^^19^^,^^24^.

Several sleep disorders were identified, but in an inconsistent way.The disorders appeared in one, two or at most three studies - out of eight articles. To synthesize the data, in general, the main problems pointed out were related to the onset of sleep (bed time resistance, sleep onset delay, the difficulty arising in the morning, difficulty with sleep onset, restlessness during sleep and sleep latency - terms used in the studies)^17^^,^^19^^-^22^,^^24^.

Three studies did not identify differences in the objective measures between the groups^17^^,^^19^^,^^24^. However, one study objectively reported less duration of REM sleep and overall sleep duration^18^ (Table 2).

**Table 2:** Description of the selected studies.

Author/ Year	STROBE *check-list*	Objectives	Population and sample	Age	Method	Results
Corkum et al. (2001)^17^	13,5/14,5	To evaluate sleep parameters in children with ADHD and compare to normal children.	GADHD (n=25 - unmedicated) and CG (n=25).	7-11 years old	- CSQ-P. - Actigraphy for 7 consecutive nights: 4 parameters (total sleep duration, sleep onset, n of night awakenings and restless sleep). - Child Sleep Diary (specifically developed for this study).	- CSQ-P: GADHD >sleep duration (*p<*0.05), >bedtime resistance (*p<*0.01), difficulty with sleep onset (*p<*0.05), >difficulty arising in the morning (*p<*0.01) and restlessness during sleep (*p<*0.01).
						- Actigraphic data: there were no significant differences between the groups (p>0.05).
						- Sleep Diary: GTDAH >sleep duration (*p<*0.05) and>bedtime resistance (*p<*0.01).
Gruber et al. (2009)^18^	dez/15	To examine sleep architecture and score the problems found in children with ADHD and normal controls.	GADHD (n=15 -	7-11 years old	- Polysomnography (at home).	- GADHD: <sleep duration (*p<*0.05), <REM sleep duration (*p<*0.05), <percentage of the total REM sleep time (*p<*0.05) and >score related to insufficiency and anxiety factors in sleep (*p<*0.05).
			unmedicated) and CG (n=23).		- CSHQ.	
Choi et al. (2010)^20^	dez/14	To assesssleep characteristics in children with	GADHD (n=27 - unmedicated) and CG (n=26).	7-12 years old	- CSHQ.	GADHD: >sleep onset delay (*p=*0.027), >sleep duration (*p=*0.032), >night waking (*p=*0.006), >daytime sleepiness (*p=*0.007), >parasomnias (*p=*0.016), >and total sleep disturbance factors (*p<*0.001).
		ADHD.			- Polysomnography (1 night).	- Polysomnography: there were no significant differences between the groups.
Gruber et al. (2012)^19^	15 / 15	To determine the contributions of circadian preferences and behavioral problems to sleep onset problems of children with ADHD.	GADHD (n=26 - 48h no medication) and CG (n=49) (control).	7-11 years old	- Polysomnography (at home).	- Polysomnography: there were no significant differences between the groups.
					- CSHQ. Sleep reports: parents documented child bedtime over 4 nights.	- GADHD: >score on CSHQ difficulty with sleep onset (*p≤*0.05), >sleep duration (*p≤*0.05), >night waking (*p≤*0.001), >sleep anxiety (*p<*0.05) and >daytime sleepiness (*p≤*0.001).
					- CSHQ.	
Zambrano-Sánchez et al. (2013)^24^	11/nov	To compare the frequency of sleep disorders and executive dysfunction in children with and without ADHD.	GADHD (n=156 - unmedicated) and CG (n=111).	7-12 years old	- PSQ.	The most frequent sleep disorders were (ADHD/ Control): obstructive sleep apnea-hypopnea syndrome (69% / 63%), inadequate sleep hygiene (66% / 76%), restless legs syndrome (57% / 62%), periodic limb movement disorder (56% / 64%). All with p>0.05.
Lee et al. (2014)^21^	13 / 13	To examine neurocognitive functions and nocturnal sleep parameters in patients with ADHD.	GADHD (n=37 unmedicated boys) and CG (n=32).	7-12 years old	- Actimetry for 72 hours during weekdays, evaluating 5 parameters	- GADHD: > sleep latency (16.2±19.7min versus 6.6±6.2 min →*p=*0.01), > wake after sleep onset (57.4±23.2min versus 30.7±13.7min →*p<*0.001), >fragmentation index (16.7±4.5 versus10.3±4.4 →*p<*0.001).
					(total sleep time, sleep latency, sleep efficiency, wakefulness after sleep onset, and fragmentation index).	
Akinci et al. (2015)^22^	14/nov	To evaluate the macrostructure and microstructure of sleep in children with ADHD.	GADHD (n=28 - unmedicated) and CG (n=15).	GADHD: 8-12 years old	- Pittsburgh Sleep Quality Index.	- GADHD: >chronic sleep onset insomnia
				CG: 9-13 years old.	- Modified Epworth Sleepiness Scale.	(*p<*0.001), >sleep talking (*p=*0.001), >daytime sleepiness (*p=*0.018), worse sleep quality (*p=*0.002), >sleep latency (*p=*0.001) and <sleep efficiency (*p<*0.001).
					- Questionnaire of sleep habits and parassomnias (answered by parents).	- GADHD macrostructure: >percentage of REM sleep, >periodic limb movement index (*p=*0.024).
					- Video Polysomnography (1 night).	- GADHD microstructure: < percentage of cyclic alternating pattern time to NREM sleep time (*p=*0.031) and N2 (*p=*0.019), reduced A1 index in N2 (*p=*0.033) and shorter sequences mean duration (*p=*0.044).
Durmuş et al. (2017)^23^	16,5 / 13	To examine the chronotype preferences in children with ADHD in the context of sleep disorders.	GTDAH (n=52 - unmedicated: 73% combined, 19% inattentive, 8% hyperactive-impulsive) and CG (n=52).	7-12 years old	- CSHQ.	- CSHQ (the children were split into 3 subgroups - morning, intermediate and night according to the cut-off): In children with ADHD the commitment of sleep was 33,5% in the morning subgroup, 86,2% in the intermediate subgroup and 100% in the night group; while in the control group these values were 50%, 86,2% and 76,9%, respectively. GADHD: > score in CSHQ (*p<*0.01).
					- Parameters of sleep/awake: Chronotype questionnaires for children.	- GADHD: positive correlation between the highest scores at night and the total scores in resistance to the sleep duration (*p=*0.005), breathing problems during sleep (*p=*0.027) and daytime sleepiness (*p=*0.001).

The disturbances identified in the studies were night walking^19^^,^^24^, parasomnias^19^, obstructive sleep apnea^23^, restless legs syndrome^23^, periodic limb movement disorder^21^^,^^23^, sleep talking^21^, breathing problems^22^ in ADHD group. In addition, higher scores of daytime sleepiness^21^^,^^22^^,^^24^ and in Children’s Sleep Habits Questionnaire (CSHQ)^19^ were presented in the ADHD group compared to the group control. The individual results of each study, as well as the methodological scores, are presented in Table 2.

## DISCUSSION

The present systematic review aimed to verify studies comparing sleep disorders in 7 to 12-year-old children with and without an ADHD diagnosis and provides an update to other already publishedreviews. Furthermore, we took precautions to control the intercession of other associated comorbidities, which included non-medicated children in the data collected, as well as analysis of both objective and subjective measures.In general, it was observed that children with ADHD present greater sleep problems when compared to children without the diagnosis.

The sleep evaluation can be conducted through objective or subjective measures. Polysomnography is one of the objective measures. It is conducted in the laboratory for an entire night and it is considered the gold standard method for the diagnosis of sleep disorders. Another method - a lower cost one - that also assists the sleep analysis is the actigraphy, which allows the evaluation of the sleep-wake cycle.

When compared to polysomnography, the actigraphy presents a 0.8-0.9 reliability coefficient in the evaluation of on parameters such as total time asleep, total time awake, number of awakenings and sleep latency^25^. That is a great alternative, although it does not replace the polysomnographic study^25^. On the other hand, the subjective measures include questionnaires, considered a low-cost measure, easily applied and with a short evaluation period.They can be applied alone, but also complementarily used with objective measures^23^. The selected studies in this review adopted both forms of evaluation, and the *Children’s Sleep Habits Questionnaire* (CSHQ) was primarily used^18^^,^^19^^,^^22^^,^^24^. 

The meta-analysis of Cortese et al.^6^ also included studies with subjective and objective measures. It points out that when analyzing subjective measures, children with ADHD had significantly more difficulties related to sleep when compared to children without the disorder: they had higher bedtime resistance (z=6.94, *p*<0.001), more sleep onset difficulties (z=9.38, *p*<0.001), night awakening (z=2.15, *p*=0.031), difficulties with morning awakening (z=5.19, *p*<0.001), sleep disordered breathing (z=2.05, *p*=0.040) and daytime sleepiness (z=1.96, *p*=0.050). However, in the objective measurements, several parameters did not differ between groups. Despites that, some parameters, such as sleep onset latency (z=3.44, *p*=0.001), sleep hours (z=2.43, p=0.015) and apnea-hypopnea index (z=3.47, *p*=0.001) were significantly higher in children with ADHD, as well as lower sleep efficiency (z=2.26, *p*=0.024), true sleep time on actigraphy (z=2.85, *p*=0.004) and average time to fall asleep during the Multiple Sleep Latency Test (z=6.37, *p*<0.001). Our results are still somewhat inconsistent, corroborating some studies analyzing sleep through objective measures, which also did not register differences between the groups^17^^,^^19^^,^^24^.

Regarding the most evidenced sleep disorders in the current study, there are bedtime resistance, difficulties with the sleep onset, restless sleep, increased sleep latency, nocturnal awakening, and daytime sleepiness. Cortese et al.^6^ also found these results in their meta-analysis and proposed that difficulties initiating sleep and bedtime resistance could be justified by the greater initial sleep latency time in children with ADHD. In this context, objective studies using video monitoring indicate that children with ADHD present increased sleep latency, decreased REM sleep percentages, and increased nocturnal activity^18^^,^^20^^,^^21^^,^^26^.

The relationship between sleep disorders and types of ADHD (hyperactive, inattentive and combined) is another analysis that has been discussed. The only study in this review that analyzed this correlation was Zambrano-Sánchez et al.^24^, which observed a significant correlation between the frequency of periodic limb movement disorder and the frequency of the combined type of ADHD (0.78); and between the frequency of inadequate sleep hygiene and the frequency of the hyperactive and combined types of ADHD (0.65).

Similarly, other studies correlated the subtypes of ADHD with the presence of restless legs syndrome through video-polysomnography, a sleep disorder characterized by the involuntary need to move the legs. A positive correlation was found between the presence of the syndrome and hyperactive behavior^27^^-^29, which corroborates the findings of this review.

Therefore, the literature points out that children and adolescents with ADHD are significantly more impaired regarding sleep efficiency than those without the diagnosis in most of the subjective measures and some of the objective measures of sleep^6^. These results establish the basis for future evidence-based guidelines on the management of sleep disorders in children with ADHD^6^, in order to improve their quality of life.

There are findings suggesting that the relationship between ADHD and sleep problems is bidirectional. In other words, sleep disorders can generate and/or exacerbate the inattention and hyperactivity symptoms, but on the other hand, these symptoms and/or the medications used to treat them may trigger or worsen some sleep disorders^30^. Stimulant medication is the most common treatment of ADHD, which increase and maintain alertness. A recent meta-analysis showed that children and adolescents had worse sleep on stimulant medications, as longer sleep latency, shorter sleep duration, and worse sleep efficiency^31^. This explanation may justify why all the studies included in this review only evaluated children outside the drug effect. However, other studies have shown that pharmacological treatment for ADHD usually has a benefic effect on sleep, which may facilitate and improve the quality of sleep in some children with ADHD^32^^-^34.

Finding out that children with ADHD, when compared to those without the diagnosis, present more sleep problems, as evidenced in present review, is extremely important for health professionals in order to signal the need for a broader assessment, including analysis of sleep, since sleep problems can aggravate the symptoms of ADHD, and from that assessment, better target the treatment and obtain better results.

In comparison with Cortese’s meta-analysis, the current systematic review has the following main strengths: to have included only studies that evaluated children from 7 to 12 years, which minimizes the influence of puberty in the analysis and indicate that in this portion of the child population with ADHD presents more sleep disturbances than children without ADHD. In the current review we present results from recent studies which, in addition to reinforcing the data already presented^6^, add important information to the literature, such as higher rates of restless leg syndrome in children with ADHD^24^ not previously presented.

As a limitation of this review, the ineligibility of studies to conduct data synthesis through meta-analysis due to the great variability, not only of the objective and subjective tools applied in the evaluation of sleep, but also of the methodology used in the application of these instruments (example: evaluation of the actigraphy for 7 days in a study *vs.* 72 hours; polysomnography in clinics *vs.* polysomnography at home) and in presenting the results. Due high heterogeneity of studies makes it inappropriate to summarize measures. As a further suggestion, it would be interesting for the studies to standardize the instruments and protocols for sleep analysis in children with ADHD, through more objective and sensitive measures to all dimensions of sleep, to facilitate the analysis of the outcomes and to enable meta-analysis elaboration.

The studies analyzed in this review show that, in general, children with a diagnosis of ADHD present more sleep disturbances when they are compared to those without the diagnosis. These disorders were diverse, yet inconsistent among the surveys. After inclusion and exclusion criteria were applied, we could only include eight studies in this review, which emphasizes the need for further research investigating sleep parameters in the context of ADHD.
